# The *portfolio effect* cushions mosquito populations and malaria transmission against vector control interventions

**DOI:** 10.1186/s12936-018-2441-z

**Published:** 2018-08-10

**Authors:** Gerry F. Killeen, Thomas E. Reed

**Affiliations:** 1 Environmental Health and Ecological Sciences Department, Ifakara Health Institute, Ifakara, United Republic of Tanzania; 2 Vector Biology Department, Liverpool School of Tropical Medicine, Pembroke Place, Liverpool, L3 5QA UK; 3 School of Biological, Earth and Environmental Sciences, University College Cork, Western Road, Cork, Republic of Ireland

**Keywords:** Malaria, *Plasmodium*, *Anopheles*, Mosquito, Vector control, Elimination, Ecology

## Abstract

**Background:**

Portfolio effects were first described as a basis for mitigating against financial risk by diversifying investments. Distributing investment across several different assets can stabilize returns and reduce risks by statistical averaging of individual asset dynamics that often correlate weakly or negatively with each other. The same simple probability theory is equally applicable to complex ecosystems, in which biological and environmental diversity stabilizes ecosystems against natural and human-mediated perturbations. Given the fundamental limitations to how well the full complexity of ecosystem dynamics can be understood or anticipated, the portfolio effect concept provides a simple framework for more critical data interpretation and pro-active conservation management. Applied to conservation ecology purposes, the portfolio effect concept informs management strategies emphasizing identification and maintenance of key ecological processes that generate complexity, diversity and resilience against inevitable, often unpredictable perturbations.

**Implications:**

Applied to the reciprocal goal of eliminating the least valued elements of global biodiversity, specifically lethal malaria parasites and their vector mosquitoes, simply understanding the portfolio effect concept informs more cautious interpretation of surveillance data and simulation model predictions. Malaria transmission mediated by guilds of multiple vectors in complex landscapes, with highly variable climatic and meteorological conditions, as well as changing patterns of land use and other human behaviours, will systematically tend to be more resilient to attack with vector control than it appears based on even the highest quality surveillance data or predictive models.

**Conclusion:**

Malaria vector control programmes may need to be more ambitious, interpret their short-to-medium term assessments of intervention impact more cautiously, and manage stakeholder expectations more conservatively than has often been the case thus far.

## Background

Conservation biologists have recently adopted the *portfolio effect* concept from economics [1], to guide their thinking in relation to ecosystem conservation [2]. The implications of such simple probability theory for financial investments are rather obvious and now widely accepted: diversification stabilizes investment portfolios, thereby reducing risks of catastrophic losses [1]. Distributing investment across several different assets can stabilize returns and reduce risks by statistical averaging of individual asset dynamics that often correlate weakly or negatively with each other [1].

The same simple probability theory [1] is equally applicable to complex ecosystems, which are buffered against natural and human-mediated perturbations by biological and environmental diversity [2]. Rather than rely on prescriptive model predictions, the uncertainties of which are determined by fundamental limitations to how well the full complexity of ecosystem dynamics can be understood or anticipated, the portfolio effect concept provides a simple framework for more critical data interpretation and pro-active conservation management [2]. Merely understanding the portfolio effect concept informs management strategies emphasizing identification and maintenance of key ecological processes that generate complexity, diversity and resilience against inevitable and often unpredictable perturbations [2].

## Implications for malaria vector control and surveillance

The implications of the portfolio effect concept should also be considered when interpreting malaria vector surveillance data and the predictions of simulation models fitted to them. When considering how uncertain models might be, it is important to distinguish between the likely causes of unbiased imprecision and systematic inaccuracy. The portfolio effect introduces the latter: by design, mathematical models are deliberately less complex than the biological system they are intended to mimic [3–5], and no dataset can capture all the different circumstances a real biological system experiences. There is, therefore, an inevitable tendency for mathematical models to underestimate the complexity and associated resilience of natural biological systems. Expressed in simple interpretational terms, mosquito populations and malaria transmission will tend be more resilient against control efforts than face-value interpretation of data or predictive mathematical models suggest.

The ubiquitous and extreme heterogeneities of vector density and vectorial capacity that occur across remarkably fine geographic scales have long been recognized as crucial factors underpinning the notorious intransigence of malaria transmission to intervention efforts [6–8]. However, beyond heterogeneities of vector density resulting in local foci where transmission is far more intense and stable than the landscape-wide average, vector biodiversity and heterogeneities in the environments they live in create portfolio effects that diversify the properties of malaria transmission.

Modelling analyses that incorporated heterogeneities of mosquito behaviour were centrally important to the illustrations of how *residual* malaria transmission [9–12] persists so robustly in Africa after scale-up of indoor residual spraying (IRS) [13, 14]. Since then, a variety of models have been used to illustrate this same point [15–30]. However, no existing model captures the full range of all relevant mosquito behaviour in real transmission systems with biodiversity spanning dozens of vectors [31], several of which may occur in any given setting. While such models can be improved, progress towards more realistic representations of complex real-life vector systems will be limited by data and understanding for the foreseeable future [5, 32]. In the meantime, it may be prudent to bear in mind the following rule of thumb: the more diverse and variable the life histories of malaria vectors are, the less likely it is that any given vector control approach with eliminate all the malaria transmission they mediate.

For example, the more mosquito species a malaria parasite can use as a vector, the higher the probability that one or more of those species will become resistant to any given insecticide compared to a situation where only a single vector species is involved. Given that there is a stochastic element to resistance evolution, the more vector species are present, the more likely that at least one of them will become physiologically resistant to insecticides and continue to mediate transmission despite high coverage of long-lasting insecticidal nets (LLINs) and/or IRS. For example, while *Anopheles gambiae* has been greatly reduced in numbers across many parts of Africa following scale-up of pyrethroid-based LLINs [33, 34], highly pyrethroid-resistant *Anopheles funestus* [35] may persist and mediate intense transmission [36].

Also, differences in the behaviour of mosquito species have long been known to render malaria transmission frustratingly resilient against attack with IRS [14, 37, 38]. Behaviourally selective vector control interventions, such as LLINs and IRS, have successfully eliminated entire populations of some of the world’s most important malaria vectors, such as *An. gambiae* and *An. funestus* in Africa, *Anopheles punctulatus* and *Anopheles koliensis* in Oceania, or *Anopheles darlingi* in South America. However, elimination of malaria transmission remains elusive in most settings because mosquito species persist which are less efficient vectors but also exhibit outdoor resting and feeding behaviours that are far less vulnerable to these indoor-targeted approaches [9, 10, 28, 34, 39].

Furthermore, fine-scale environmental variations in the relative abundance and availabilities of essential blood host and resting site resources can drive huge variations in the behavioural choices that mosquitoes exhibit in different parts of a given landscape. Taking *Anopheles arabiensis* as an African example of an important vector of residual malaria transmission that exhibits notoriously plastic feeding behaviours, the proportion of indoor-feeding mosquitoes that rest indoors until the following morning can vary by two orders of magnitude [40]. More tellingly, *An. arabiensis* can exhibit both extremes of feeding predominantly on either people or cattle, even in different family compounds within the same small village [41, 42].

**Fig. 1:**
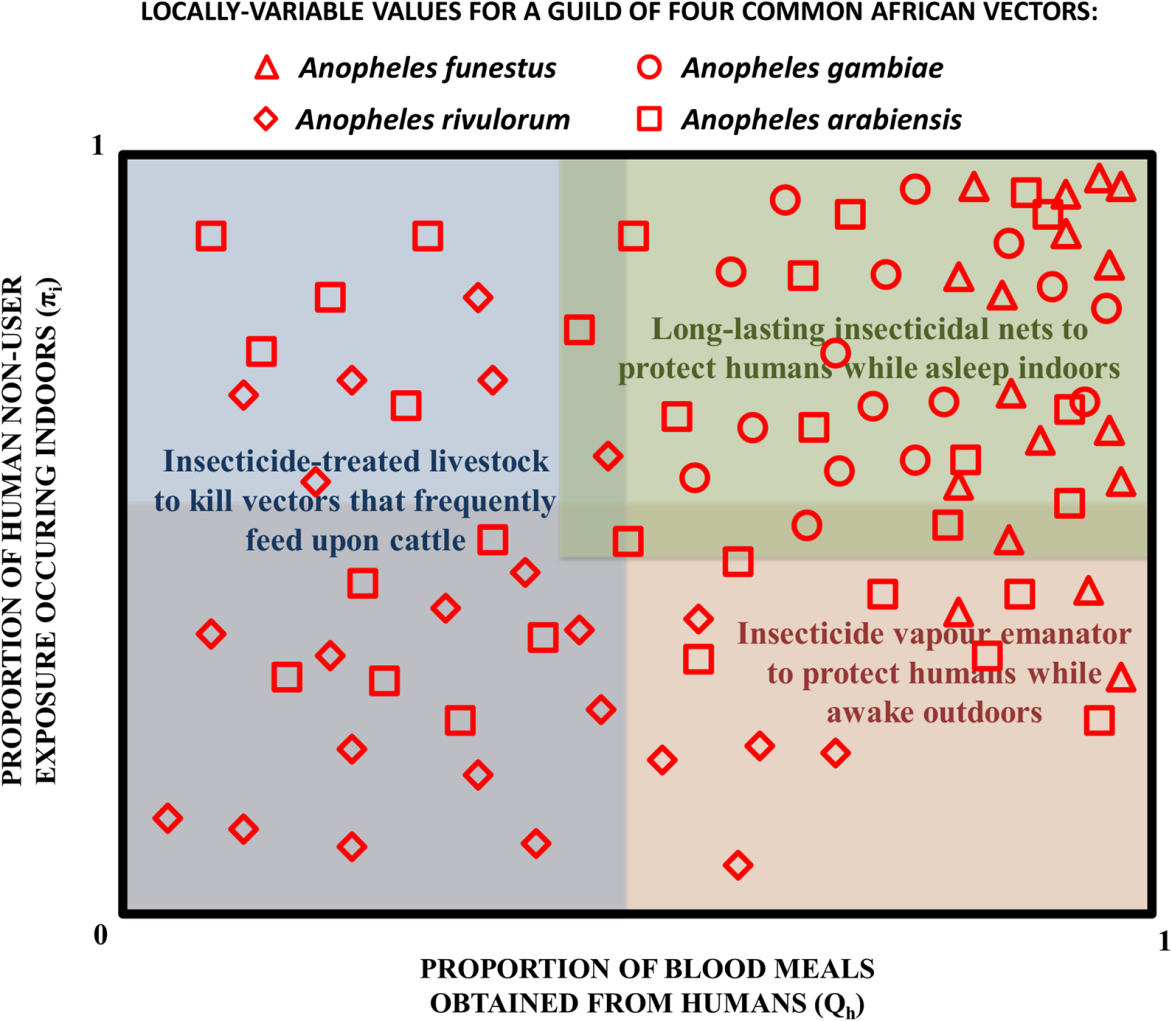
A schematic illustration of how a hypothetical but typical guild of four common African malaria vectors may span the full range of behavioural preferences for biting humans indoors versus outdoors, and biting humans versus animals. While *An. funestus* has a very strong preference for humans [28, 42], it is capable of biting early in the evening or late in the morning when humans are active and exposed outside the protective reach of long-lasting insecticidal nets [66–70]. *Anopheles gambiae* has a slightly less strict preference for feeding upon humans [28, 42] and can also feed outdoors at dawn and dusk to some degree in some locations [17, 71]. *Anopheles arabiensis* is notoriously phenotypically plastic in its expression of both behaviours, spanning a very wide range of human blood indices [28, 42] and often biting outdoors in the early evenings in settings where effective indoor vector control has been implemented [17, 72]. While *Anopheles rivulorum* typically prefers to feed upon animals, and tends to be most active at dusk and dawn, it is nevertheless a vector of malaria in its own right, contributing significantly to residual transmission in some settings [51, 73, 74]

Consequently, there is no single correct choice amongst insecticidal vector population suppression interventions that target this species when they either attack humans while asleep indoors (LLINs/IRS), when they attack people outdoors (vapour-phase insecticides or insecticide-treated clothes), or when they attack cattle (veterinary formulations of systemic insecticides, often referred to as endectocides) [29] (Fig. 1). Because of such fine-scale heterogeneities in the behavioural choices such phenotypically plastic species make in response to variations they encounters in their environments [43, 44], quite large local gaps in biological coverage [20] inevitably arise regardless of which of these approaches is chosen [29]. Given that such extreme heterogeneities of behaviour an occur within even a single species over distances of metres rather than kilometres [43, 44], it will never be possible for control programmes to map them out across national scales with sufficient resolution to enable accurate, biologically relevant targeting of each individual intervention [29].

The diversity of behaviours expressed by a single vector species through phenotypic plasticity is further exacerbated by the fact that they usually co-exist alongside other vectors with different behavioural preferences (Fig. 1). Such guilds of multiple vectors may span a remarkably wide range of behavioural phenotypes, and the African scenario presented in Fig. 1 is far less biodiverse than many settings in southeast Asia [9, 45–47]. It will therefore be necessary to design packages of complementary vector control interventions based on the range of behaviours observed in nationally representative surveys, rather than their mean values, so that these intervention combinations are broadly applicable and robust to local variations in the behaviours targeted by each component control measure [29]. Such a multi-intervention approach would also help address the urgent need to implement insecticide resistance management strategies [48], by exploiting multiple interventions that allow different, complementary insecticide classes to be deployed as combinations delivered through distinct products.

**Fig. 2:**
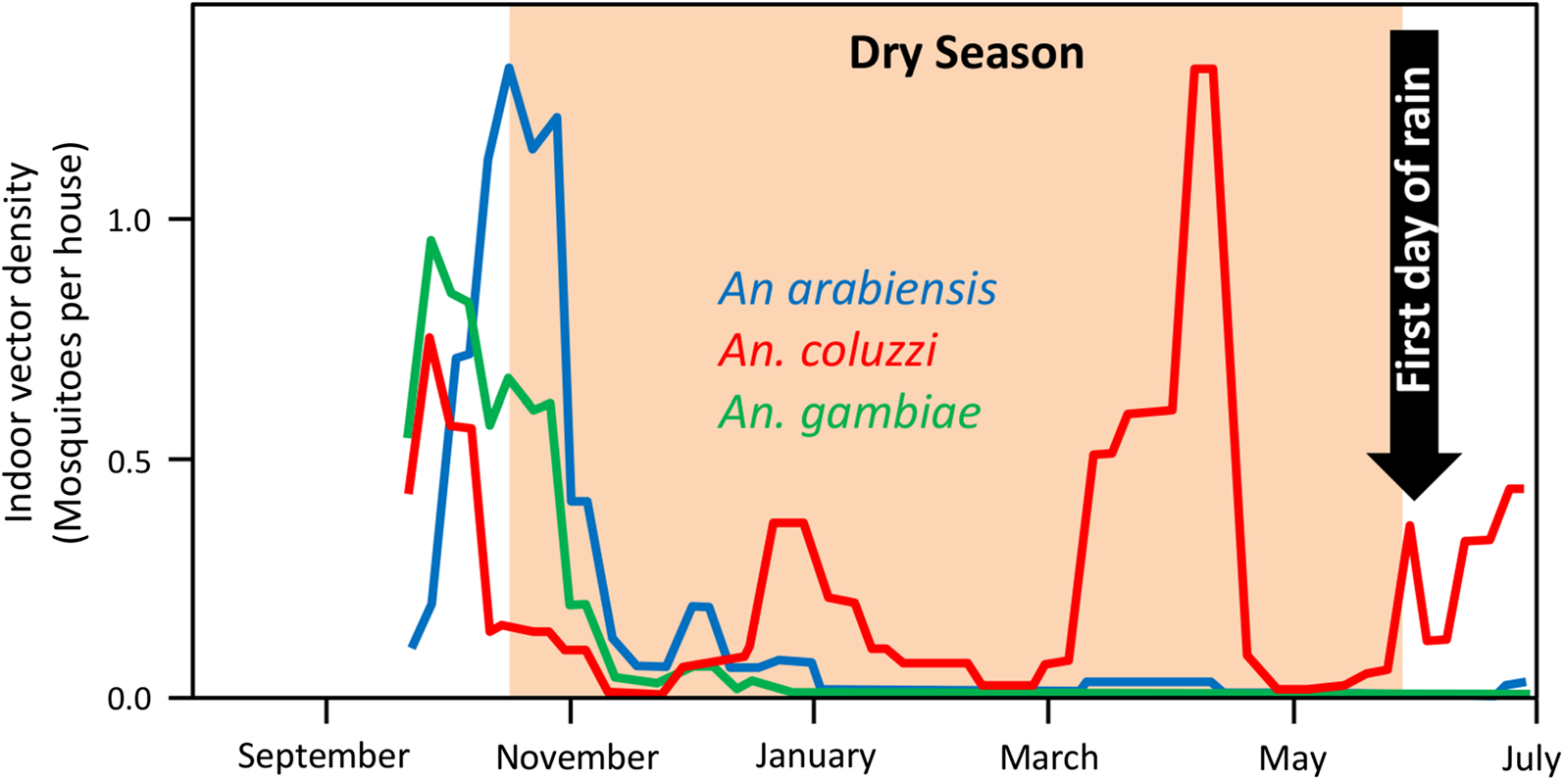
An example of how the seasonality of three sympatric sibling vector species from a single complex can exhibit very different seasonality patterns in the same location. In this case *An. arabiensis*, *An. coluzzi* and *An. gambiae* in the Sahel of Mali, redrawn from Ref. [58] for the 2007 to 2008 season

Complex interactions between landscape hydrology, weather patterns and vector biology also generate diversity in the characteristics and distribution of aquatic habitats that one or more vector species utilize, as well as diversity in the seasonality in their population dynamics. As a result, the probability that at least one habitat type will occur that is difficult to target with larval control is increased relative to stereotyped expectations based on any single species. For example, much has been written about the opportunities and obstacles to targeting members of the *An. gambiae* complex based on the stereotyped assumption that they predominantly breed in clean, sunlit, rain-fed “pools and puddles” [49], when the reality is that these species exhibit considerable plasticity in their oviposition behaviour and often do so in impressive style [50, 51]. *Anopheles gambiae* sensu lato larvae have been repeatedly documented in atypical, non-preferred, often cryptic habitats such as tree holes, borrow pits, the vegetated fringes of fast-flowing rivers, and water storage containers, especially during the dry season when options are otherwise limited [50, 51]. Critically, the larval ecology of this complex is notoriously variable between and within species of the complex [52–54], so the exact survival strategy exhibited in any given location is idiosyncratic and essentially impossible for even experts to reliably predict [55, 56]. Variations in larval ecology between two or more vectors, or between the seasonal dynamics of different habitats in the same ecosystem, also create diversity of seasonality that provides refuges against vector control interventions. For example, a small sub-set of locations with high water tables that support permanent lakes, ponds and swamps, or perennial rivers and streams create local conditions where transmission occurs all year round. In Africa south of the Sahara, such hydrological conditions create a niche for *An. funestus* [51], perhaps the most efficient vector of malaria in the world and now highly resistant to pyrethroids all across its distribution [35]. In some locations, transmission peaks well into the dry season when receding water bodies create abundant habitat. Perhaps the most dramatic historical example is the dry season malaria epidemics in Sri Lanka caused by *Anopheles culicifacies* breeding in dried-out river beds [57]. Where two or more vector species exhibit seasonal peaks of transmission at different times of the year, or even where multiple habitat types for a single vector species exhibit different seasonal patterns, transmission seasonality is diversified and therefore becomes more resilient to any transmission control measure applied discontinuously. Even within the *An. gambiae* complex, *Anopheles coluzzii* can aestivates through the Sahelian dry season before re-awakening a month or two in advance of the first rains, approximately 6 months apart from its far more rain-dependent siblings, *An. arabiensis* and *An. gambiae* (Fig. 2) [58]. There is therefore no perfect time of year to implement IRS, seasonal larviciding or even mass drug administration campaigns in real landscapes, and interannual variability introduces further scope for missing a moving target. We must settle for averaged and therefore imperfect timing optima for any seasonally implemented intervention. It is, therefore, important to take a realistic, pragmatic view of what impact may reasonably be expected from any given intervention approach, no matter how optimally seasonal delivery is timed based on averaged seasonality trends.

## Conclusions

All these examples of the complexities that bolster malaria transmission against vector control interventions can be bamboozling and distract from very simple common principles that underlie them all. Malaria parasite populations that typically spread their reproductive bets across two or more vectors with different behaviours, ecological niches or seasonality dynamics will systematically be more difficult to eliminate than in the rare settings with a single vector species. Furthermore, where individual vector species spread their own reproductive bets across multiple aquatic habitat types, resting sites or blood sources, this creates refugia that limit the impact of any given vector control measure applied in any given time and place. And no matter how much detail we try to capture in mathematical models of vector biology and malaria transmission, they will always under-represent the full complexity and diversity of those interactions, so they are biased towards underestimating the resilience of malaria transmission against vector control. Whatever the shape of the expected response curve following introduction of a new vector control measure, the portfolio effect will tend to flatten it out to some extent. Given that the magnitude of such portfolio effects are unknown in any given location, the only sensible way to deal with their implications is to emphasize the need for cautious interpretation of entomological surveillance data, as well as simulation models extrapolating these trends into the future.

**Fig. 3:**
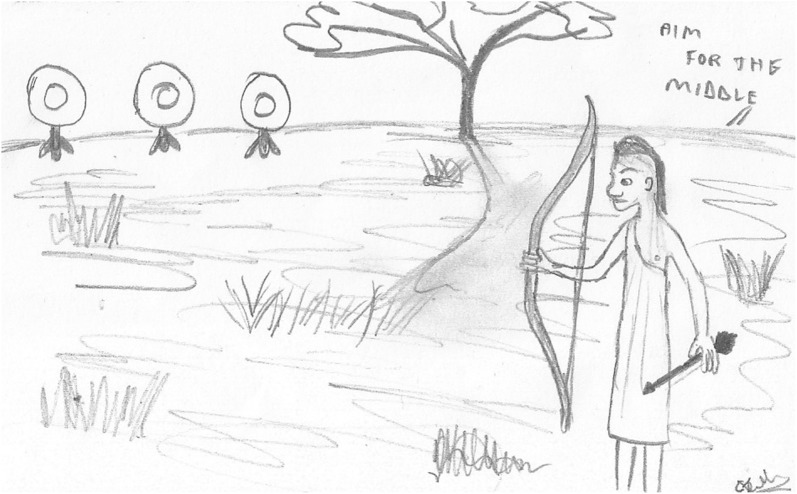
A humorous representation of the implications of the portfolio effect for practitioners and advocates for rationally targeted malaria vector control. kindly drawn by Ms. Eleanor Campos Killeen

While several recent simulation models suggest good reasons for optimism going forward [30, 59, 60], harsh lessons [39, 61, 62] learned from historical mistakes [63, 64] and awareness of ubiquitous but unquantifiable portfolio effects within malaria transmission systems both merit careful consideration. Looking ahead, it will be critically important to manage the expectations of stakeholders in malaria vector control and product development more conservatively and responsibly than has often been the case thus far [39, 61–65]. While there are and always will be limitations to knowledge of malaria transmission and control, it is the maturity with which that knowledge is applied that will “determine whether we are living in an era of hubris or indeed in an age of eradication” [5] (Fig. 3).

## Abbreviations

LLINs: long lasting insecticidal nets
IRS: indoor residual spraying
